# The economic returns to citizenship: Evidence from a meta-analysis

**DOI:** 10.1073/pnas.2617804123

**Published:** 2026-09-28

**Authors:** Jens Hainmueller, David D. Laitin

**Affiliations:** ^a^ Department of Political Science, Stanford University, Stanford, CA 94305; ^b^ Immigration Policy Lab, Stanford University, Stanford, CA 94305

**Keywords:** citizenship, naturalization, immigrant earnings, meta-analysis, selection bias

## Abstract

Citizenship is widely summarized as producing a “naturalization premium” for immigrants, but naturalization is selected: immigrants who naturalize differ in language, residence, employment, and motivation. We provide a quantitative meta-analysis of citizenship effects on immigrant earnings, synthesizing 60 preferred estimates from 31 studies. How a study separates the effect of citizenship from selection into citizenship—its identification strategy—explains more variation in estimates than destination country, citizenship regime, or any other study feature we code. Averaging published estimates with a multilevel random-effects model yields a pooled premium of 9.9% (95% CI 5.1 to 14.9), and 11.0% (95% CI 5.7 to 16.6) when pooling within study-country cells. But this average conceals sharp differences across research designs. The randomized encouragement study relying on a lottery to overcome selection implies a near-zero effect of 1.9% (95% CI −1.3 to 5.3); the regression-discontinuity study yields 11.3% (95% CI 5.2 to 17.8); estimates from policy reforms and within-unit panels are positive on average but too imprecise to distinguish from either zero or the pooled average; and observational instrumental-variable estimates average 53.0% (95% CI 27.6 to 83.5). Excluding observational instrumental-variable estimates reduces the pooled premium to 6.0% (95% CI 3.8 to 8.2). Reanalyzing the randomized trial data shows the same pattern: Experimental estimates are near zero, while an observational difference-in-differences comparison based on self-selected citizenship uptake implies a 9.6% gain (95% CI 4.0 to 15.5) four years post lottery, a bias standard pretrend tests do not detect. The published citizenship premium is positive, but its size and causal interpretation depend on research design.

## Significance

Citizenship is widely viewed as a policy lever for immigrant economic mobility, and policy and scholarly summaries often describe naturalization as improving wages and access to better jobs. Our synthesis shows why that summary needs a design caveat. Across 60 preferred earnings estimates from 31 studies, the average reported premium is about ten percent. But the most credible designs do not support a causal effect of that size. The randomized study is near zero, the regression-discontinuity study is positive, reform and panel estimates are positive on average but too imprecise to distinguish from zero or the average, and observational instrumental-variable estimates are largest and unstable. Citizenship may matter, but observational averages should not be used as causal forecasts for policy.

Naturalization changes both the rights immigrants hold and the signals they send in the labor market. By granting formal membership, citizenship can remove barriers to work and mobility, stabilize legal status, and alter employers’ beliefs about permanence and host-country skills. Together, these channels motivate the expectation of an earnings premium, an expectation that has become the dominant summary of the literature. The National Academies’ report on immigrant integration concluded that U.S. citizenship “improves employment outcomes, wage growth, and access to better jobs” (1, p. 165). OECD and subsequent reviews likewise summarize naturalization as a route to better economic integration (2–4). Empirical estimates of this “naturalization premium” have grown substantially over four decades, coming from studies whose research designs differ in how they attempt to isolate the causal effect of citizenship: cross-sectional comparisons (5, 6), panel-data fixed effects (7, 8), policy-reform difference-in-differences (9, 10), regression discontinuities (11), instrumental variables (12), and randomized encouragement (13). The literature reports a generally positive premium, but the magnitudes vary widely.

This dispersion in reported premiums matters because naturalization is endogenous to the same factors that determine earnings. Eligibility rules select on residence duration, language, criminal record, and income; application selects on motivation, information, and administrative capacity; approval selects on employment, stability, and institutional networks. Even within-immigrant fixed-effects designs are vulnerable: Prenaturalization labor-market trajectories often slope upward in anticipation of citizenship (8, 14, 15). As a result, the same population can produce very different estimated premiums depending on what comparison is drawn. Whether the published literature documents a causal effect of citizenship or a selection artifact is therefore a central empirical question for research, theory, and policy.

To answer it, we conduct a quantitative synthesis. We began with a published 53-study appendix bibliography of prior studies on citizenship and immigrant outcomes (13). We then conducted forward-citation searches and screened candidate studies through title, abstract, and full text, retaining final published versions whenever available. The earnings sample contains 60 estimand-harmonized preferred estimates from 31 studies, harmonized to a log-equivalent percent-change metric.

The causal question is counterfactual: Would the same immigrant earn more with citizenship than without it? Because we cannot observe earnings for the same person both with and without citizenship, studies must replace the missing counterfactual with an observed comparison. We refer to the source of that comparison, and the variation used to separate citizenship from selection, as the study’s identification strategy. This choice matters because strategies differ in how well they address unobserved confounders that may explain earnings differences between naturalized and non-naturalized immigrants. For example, more motivated or better informed immigrants may be both more likely to naturalize and more likely to experience earnings growth even without citizenship. Identification strategy is therefore our central moderator. We distinguish six sources of identifying variation: Randomized encouragement designs compare immigrants randomly encouraged or not encouraged to naturalize; regression-discontinuity designs compare applicants on either side of an institutional cutoff; policy or cohort reforms compare immigrants differentially exposed to changes in citizenship access; panel designs compare the same immigrants before and after naturalization; observational instrumental-variable designs use predicted citizenship status from instruments such as eligibility timing or local approval leniency; and cross-sectional designs compare naturalized and non-naturalized immigrants observed at the same time. We benchmark this focal moderator against the other extracted study features that could also shape reported premiums: treatment estimand, effect measurement window, outcome data source, earnings-sample basis, destination country, and citizenship regime measured by the 2019 MIPEX Access to Nationality score (16).

Pooling across the corpus we recover a positive average premium of approximately ten percent, but the magnitude depends sharply on identification strategy: The randomized study is near zero and precise enough to rule out premiums near the pooled average; the regression-discontinuity study is positive; reform and panel estimates are positive on average but too imprecise to distinguish from either zero or the pooled average; and observational instrumental-variable estimates are much larger and unstable. The conventional citizenship premium is therefore not a stable causal parameter; it is a design-dependent summary of a highly selected process.

## Results

### Pooled Estimates.

Across the 60 estimand-harmonized preferred earnings estimates from 31 studies (*SI Appendix*, Table S1), a multilevel random-effects model with dataset, study, and within-study sample random effects yields a pooled earnings premium of 9.9% (95% CI 5.1 to 14.9), P<0.001. A two-level model with study and within-study sample random effects and study-clustered CR2 inference gives 9.9% (95% CI 4.9 to 15.1). A study-country-collapsed model that pools to one inverse-variance summary per study-country cell yields 11.0% (95% CI 5.7 to 16.6). The three specifications agree that the published literature reports a positive average citizenship premium of approximately one-tenth of earnings. Between-estimate heterogeneity is high (total $\tau^{2}=0.0155$; $I^{2}=99.5\%$), meaning that estimates differ far more than expected from sampling error alone. Most residual dispersion is between studies ($\tau^{2}=0.0111$), with additional clustering among estimates drawn from the same within-study sample ($\tau^{2}=0.0044$). The dataset-family component is near zero ($\tau^{2}=0.0000$): Estimates from different studies that reuse the same data source are no more alike than estimates from studies using different data, once study-level differences are accounted for.

### Identification-Strategy Gradient.

**Fig. 1.**
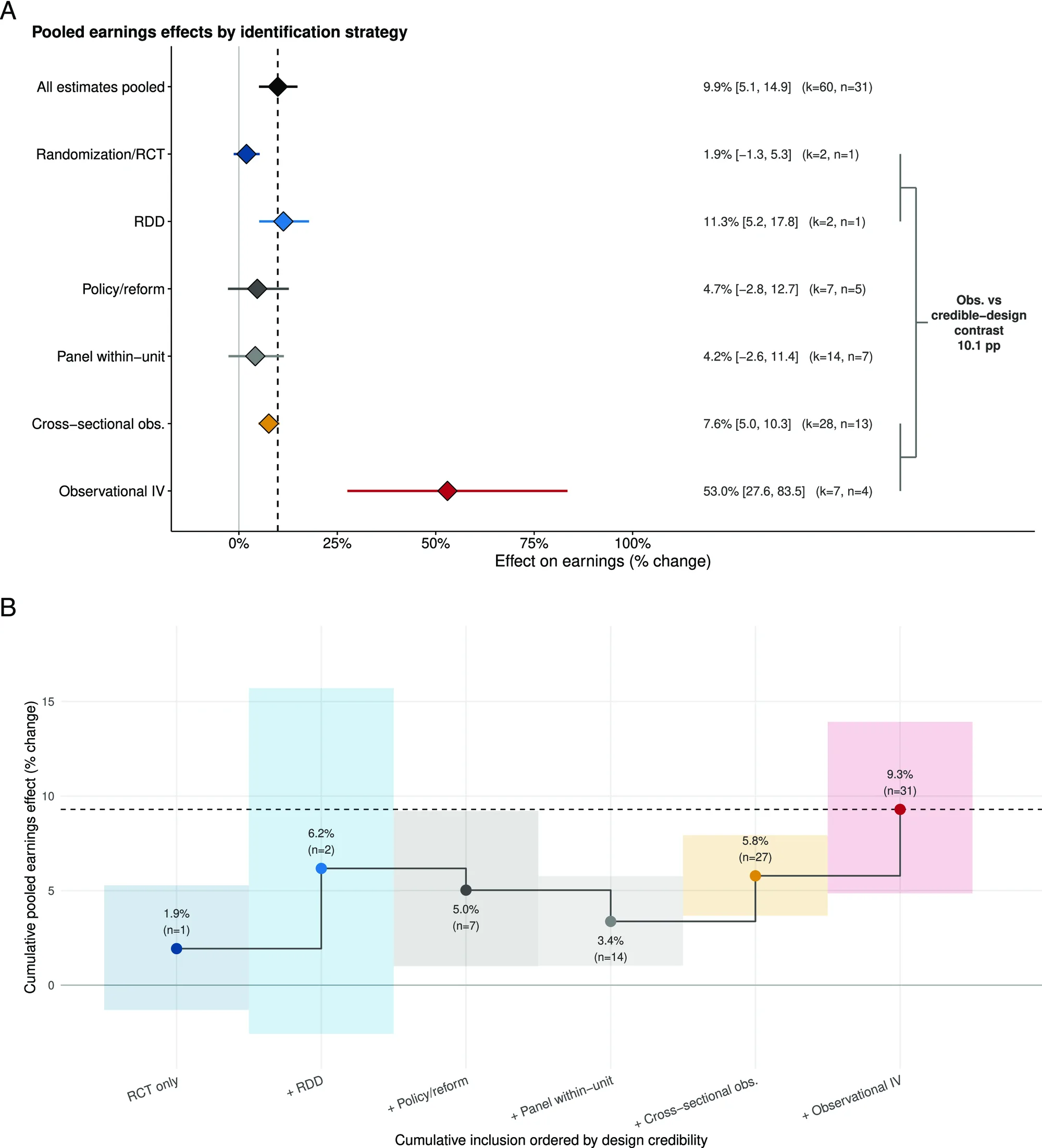
Pooled earnings effects by identification strategy and cumulative design tier. (*A*) The *Top* row shows the pooled estimate across all preferred earnings estimates; remaining rows show pooled premiums by identification strategy. Diamonds show meta-analytic point estimates with 95% CI. The vertical line marks the overall pooled estimate (9.9%). The bracket compares observational identification strategies (cross-sectional + observational instrumental-variable) with strategies that more directly target exogenous variation (randomization + regression discontinuity); the bracket value (10.1 pp) is the difference between the estimate-count-weighted means of the pooled design-cell estimates in the two blocks. In Panel (*A*), *k* = preferred estimates and *n* = studies in the row-level meta-analytic models. (*B*) Study-collapsed cumulative diagnostic. Each study is first collapsed to one effect, then study-level effects are pooled as design tiers are added from more to less credibility-dependent sources of variation. The dashed line marks the final cumulative estimate (9.3%). Panel (*B*) labels *n* as the cumulative number of study-level effects, so the count changes as design tiers enter the corpus. Because each Panel (*B*) interval pools all design tiers up to that point, it can exclude zero even when the corresponding design-specific interval in Panel (*A*) does not. The pooled premium masks pronounced design dependence: The single randomized design implies a near-zero effect, while observational instrumental-variable designs imply the largest premiums.

The pooled average citizenship effect masks pronounced design dependence (Fig. 1 and *SI Appendix*, Tables S2 and S3). The single available randomized encouragement design (13) implies a near-zero earnings effect of 1.9% (95% CI −1.3 to 5.3). A regression-discontinuity design exploiting Swiss municipal-referendum vote-share thresholds (11) yields 11.3% (95% CI 5.2 to 17.8). Policy and cohort-reform estimates pool to 4.7% (95% CI -2.8 to 12.7). Panel within-unit estimates pool to 4.2% (95% CI -2.6 to 11.4). Cross-sectional observational estimates pool to 7.6% (95% CI 5.0 to 10.3). Observational instrumental-variables estimates pool to 53.0% (95% CI 27.6 to 83.5), with the wide interval reflecting weak-instrument and exclusion-restriction concerns. The randomized interval also includes zero; what sets it apart from the reform and panel cells is what it excludes: It rules out premiums as large as the 9.9% pooled average or the 6.0% average excluding observational IV, whereas the wider reform and panel intervals remain consistent with both zero and premiums of that size. Aggregating credibility tiers, the most-credible block (RCT + RDD + policy/cohort reform; 11 estimates from 7 studies) pools to 5.0% (95% CI −0.1 to 10.4); panel within-unit estimates (14 estimates from 7 studies), which face known anticipation-effect concerns, pool to 4.2% (95% CI -2.6 to 11.4); and the observational block (cross-sectional + observational instrumental-variable; 35 estimates from 17 studies) pools to 16.8% (95% CI 7.4 to 27.0).

The pattern is consistent with positive selection into naturalization that observational designs may absorb in the citizenship coefficient. The study-collapsed cumulative diagnostic in Fig. 1*B* shows the same design dependence from a second angle, but the pattern is not a mechanical monotonic ladder. The regression-discontinuity estimate is positive, while observational instrumental-variable estimates are larger and less stable than the cross-sectional estimates. Much of the contrast is driven by two informative but unusual cells: the single randomized encouragement study at the low end and the observational-IV studies at the high end. The observational-IV category is especially instructive. Although IV designs are often placed above cross-sectional comparisons in abstract design rankings, the instruments in this literature are instruments of convenience found in observational data, not instruments created by design like the randomized fee voucher. They are indirect, heterogeneous, and often weak: eligibility timing, local approval leniency, or predicted naturalization can shift citizenship status while remaining correlated with integration trajectories, so the exclusion restriction is violated in an upward direction whenever the instrument also proxies for administrative capacity, time in host-country institutions, local integration opportunity, or other determinants of earnings growth. The arithmetic of instrumental variables amplifies such violations: Because the estimator divides the instrument’s reduced-form effect on earnings by its often small effect on citizenship, even a modest violation can make the instrumental-variable estimate more biased than the simple observational comparison it is meant to improve on. Systematic replication evidence confirms this pattern: Across 67 published instrumental-variable studies, observational instruments typically produced estimates far larger than the corresponding ordinary-least-squares results, whereas design-based instruments from lotteries and experiments showed no such inflation (17). In our corpus, too, observational IV behaves less like a clean quasi-experiment than like an unstable design family. Identification strategy matters because different comparisons recover different mixtures of selection and treatment effects; the published premium is therefore design-dependent, and the weakest observational-IV estimates should not anchor policy expectations.

### Study-Country Distribution.

**Fig. 2.**
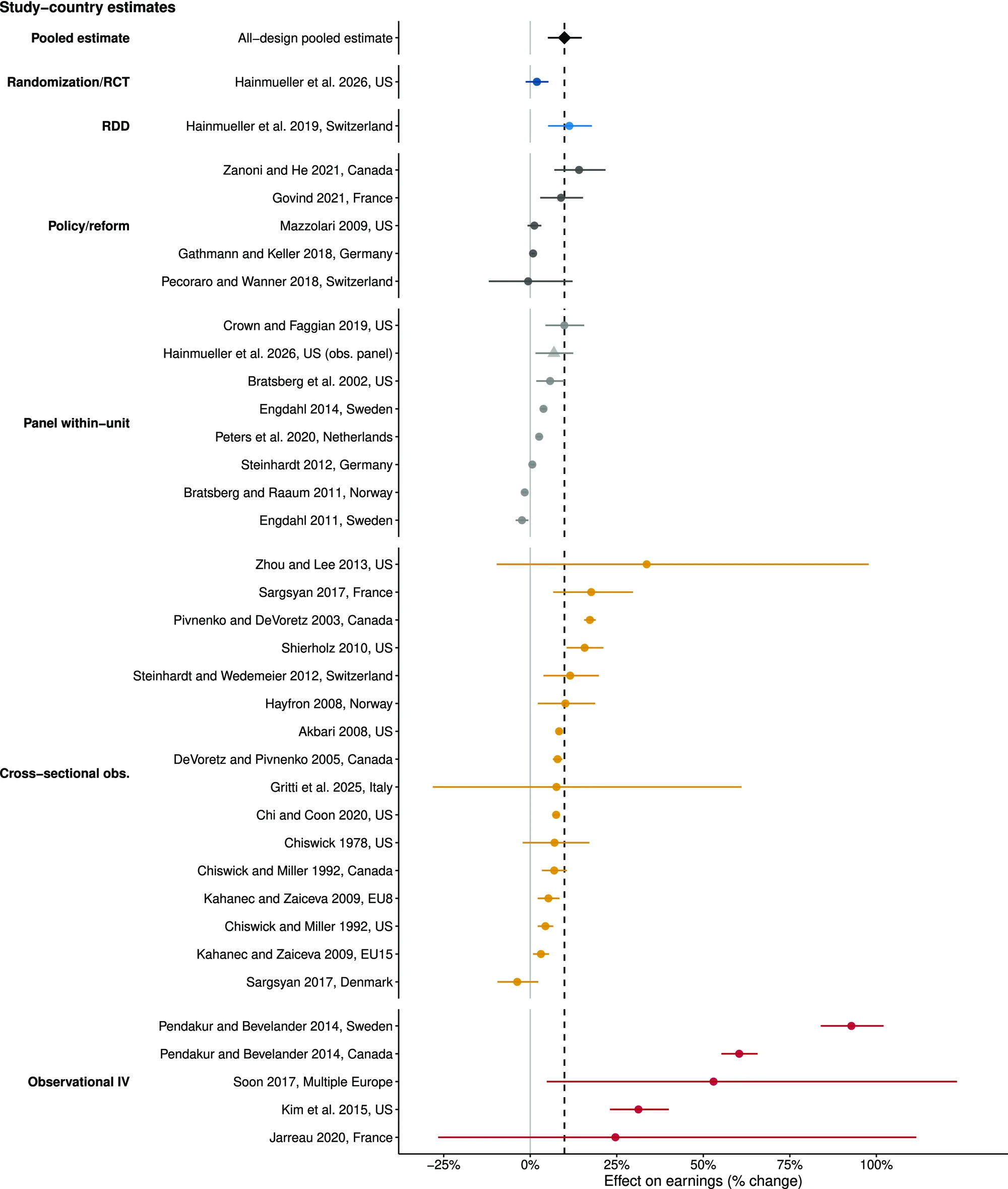
Study-country-collapsed preferred earnings estimates and observational diagnostic. The filled diamond at the *Top* shows the overall pooled estimate across all preferred earnings estimates. Filled circles show one inverse-variance-pooled preferred effect per study-country cell, with countries shown in row labels; horizontal bars are 95% CIs. The filled triangle shows the two-way fixed-effects observational panel diagnostic for the same trial sample in Hainmueller et al. (13), using control-group citizenship uptake with individual and year fixed effects, four years postlottery (*SI Appendix*, Table S6). The dashed vertical line marks the overall pooled estimate (9.9%). The positive average is not driven by any single study or country cell; the largest estimates come disproportionately from observational instrumental-variable designs.

The study-country-collapsed forest plot (Fig. 2) confirms that the pooled positive average is not driven by a single paper or a single country cell. Effects span −4% to +93%, with 31 of 35 study-country cells estimating positive effects and 24 estimating statistically significant positive effects at the 5% level. The 35 cells are generated by inverse-variance pooling duplicate estimand-harmonized preferred earnings rows within each study-destination-country pair; the count exceeds the 31-study count because four multicountry studies contribute two destination-country or regional cells. The largest estimates come disproportionately from observational instrumental-variable and weak observational specifications. The lower end of the distribution includes the randomized encouragement study and several panel within-unit studies, while the regression-discontinuity evidence is positive but comes from one institutional setting.

### Alternative Explanations.

**Fig. 3.**
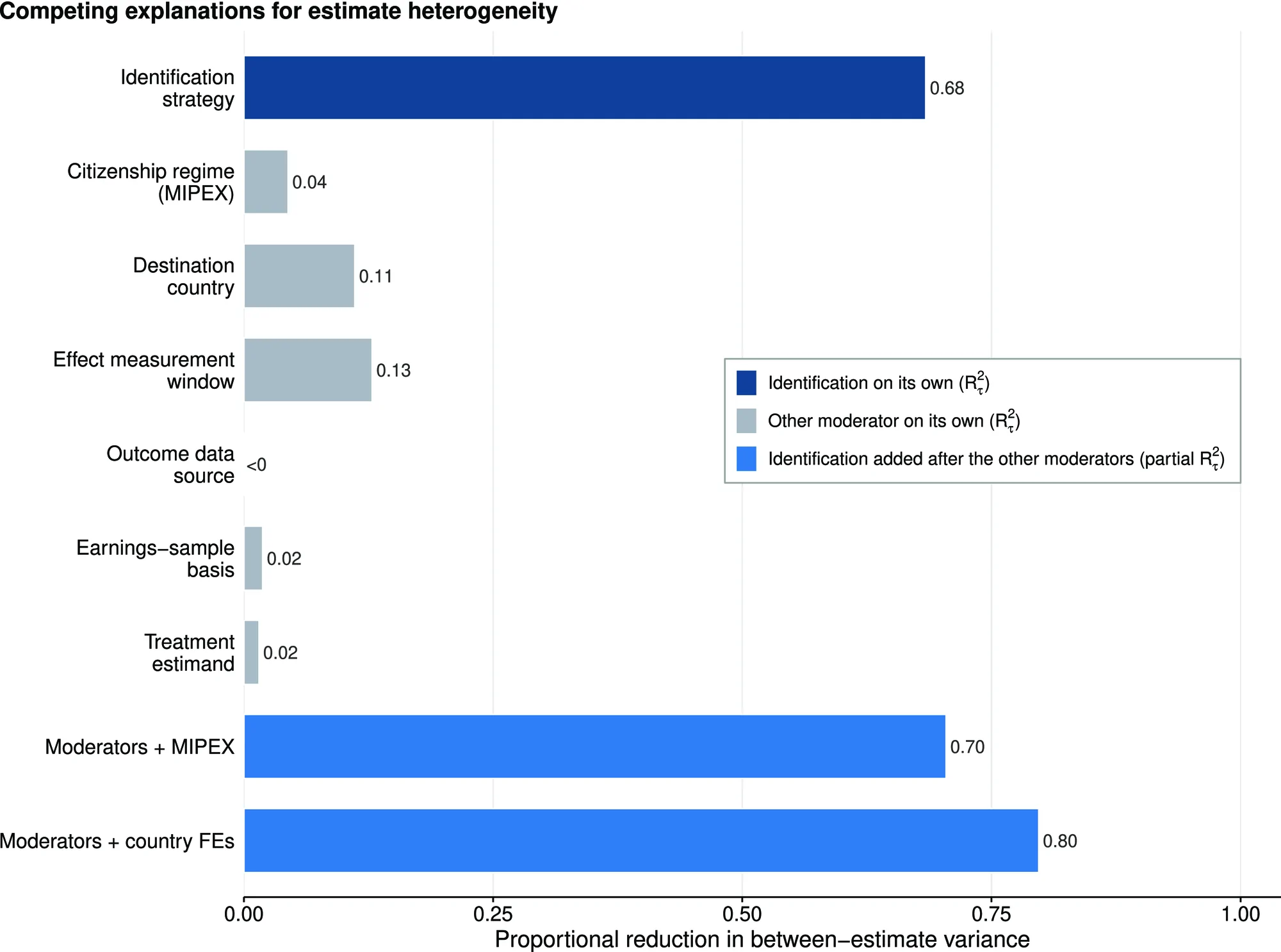
Competing explanations for heterogeneity in reported earnings premiums. Bars show the proportional reduction in between-estimate variance, $R_{\tau }^{2}$, from multilevel meta-regressions. The *Top* seven bars report each moderator separately—identification strategy, citizenship regime (MIPEX Access to Nationality), destination country, effect measurement window, outcome data source, earnings-sample basis, and treatment estimand. The *Bottom* two bars report the partial $R_{\tau }^{2}$ from adding identification strategy after the other moderators, with the citizenship regime measured by MIPEX or by destination-country fixed effects. Negative estimated reductions are plotted at zero and labeled <0; exact values appear in *SI Appendix*, Table S5. Identification strategy explains more heterogeneity than any other single moderator and adds explanatory power beyond the nonidentification controls.

The identification-strategy gradient could reflect other study features rather than the source of identifying variation. Fig. 3 tests this by fitting competing multilevel meta-regressions and comparing how much estimated between-effect variance each moderator removes relative to the null model (*SI Appendix*, Table S5). Identification strategy is the dominant one-at-a-time moderator, reducing estimated heterogeneity by $R_{\tau }^{2}=0.684$. The next largest one-at-a-time moderators are effect measurement window ($R_{\tau }^{2}=0.129$) and destination country ($R_{\tau }^{2}=0.111$). Citizenship regime, treatment estimand, outcome data source, and earnings-sample basis explain little addi tional heterogeneity on their own. The first added-after bar (moderators + MIPEX) asks the stricter question: After conditioning on citizenship regime, effect measurement window, outcome data source, earnings-sample basis, and treatment estimand, does identification strategy still matter? It does: Adding identification strategy removes a further $R_{\tau }^{2}=0.476$ of the total between-estimate variance, which amounts to a partial $R_{\tau }^{2}=0.704$ of the smaller pool of variance remaining after the controls. The second (moderators + country FEs) repeats the exercise after replacing the country-level citizenship-regime score with destination-country fixed effects; despite the heavier parameter count, identification strategy still removes a partial $R_{\tau }^{2}=0.797$ of residual heterogeneity. Among the moderators we code, identification strategy is therefore by far the most important explanation for variation in reported earnings effects, not simply a proxy for country context, citizenship-regime permissiveness, measurement window, outcome data source, earnings-sample basis, or treatment estimand.

### Within-Study Selection Check.

**Fig. 4.**
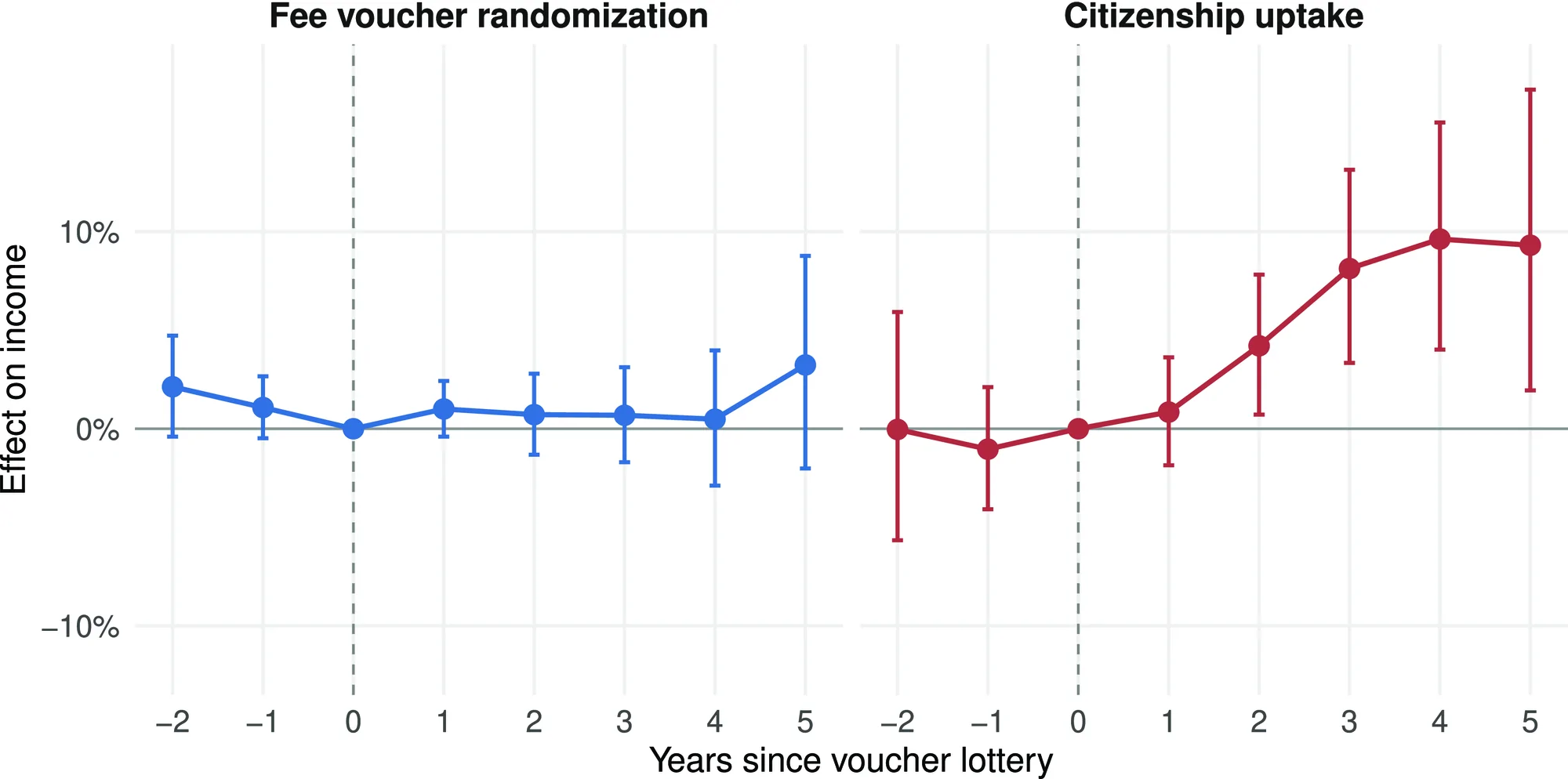
Within-study selection check in Hainmueller et al. (13). Both panels use the matched six-year credit-bureau panel and the Callaway–Sant’Anna difference-in-differences estimator for staggered treatment timing (18). Year 0 is the lottery year; the income measurement at year 0 is observed immediately before assignment. The *Left* panel estimates randomized voucher-assignment effects. The *Right* panel estimates effects of citizenship uptake among experimental control-group immigrants who were not encouraged. Points show percent effects on income with 95% CIs, transformed as $100\{\exp\left(\hat{\beta }\right)-1\}$. The *Right* panel is a selection diagnostic, not a randomized estimate. Within the same trial sample, the randomized contrast stays near zero while the selected-uptake contrast rises to 9.6%, indicating that selection into naturalization can generate much of the premium recovered by observational panel designs.

A direct within-study design-gradient check comes from Hainmueller et al. (13), where the same randomized sample can be analyzed two ways. The experimental contrast uses randomized fee-voucher assignment: Immigrants in a lottery were offered a voucher covering the naturalization application fee, which sharply increased naturalization. The observational contrast ignores the randomized assignment and uses citizenship uptake among experimental control-group immigrants who were not encouraged by a voucher, while controlling for time-invariant individual characteristics and common time shocks, as in the panel designs used in the literature. Fig. 4 applies the Callaway–Sant’Anna difference-in-differences estimator for staggered treatment timing (18) to both contrasts, using never-treated controls and aggregating group-time average treatment effects by event time. The voucher-assignment path remains near zero, while the selected postlottery citizenship path rises to about 9.6% four years after the lottery (95% CI 4.0 to 15.5). A researcher using the randomized encouragement design would therefore conclude that earnings effects are essentially zero, whereas a researcher using selected citizenship uptake in the same panel would conclude that naturalization raises earnings meaningfully. The pre-period estimates in the observational panel are close to zero, so a conventional pretrend diagnostic would not by itself reveal the postlottery selection bias in this example. This divergence suggests that time-varying selection into naturalization can explain a large part of the gains recovered by observational panel designs. *SI Appendix* reports corresponding two-way fixed-effects and fixed-effects counterfactual-imputation versions of the same diagnostic (*SI Appendix*, Figs. S2 and S3).

### Publication Bias and Significance Patterns.

**Fig. 5.**
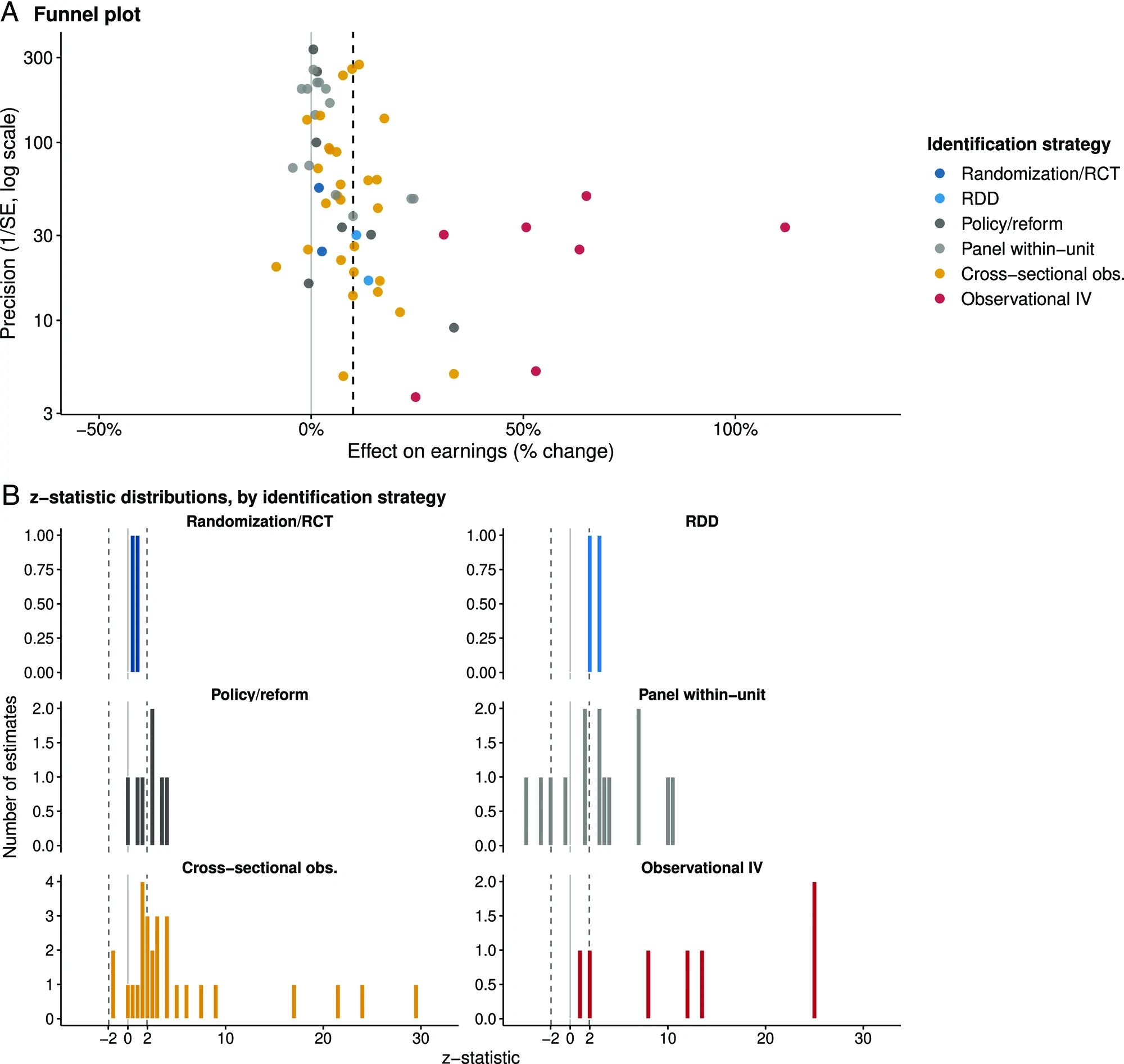
Publication bias and significance patterns by identification strategy. (*A*) Funnel plot, points colored by identification strategy. The dashed vertical line marks the pooled estimate (9.9%); the solid vertical line marks zero. (*B*) z-statistic distributions stratified by identification strategy, with vertical reference at z=±1.96. Neither diagnostic shows a strong corpus-wide publication-bias signature; the observational instrumental-variable cell skews positive, while randomization, regression-discontinuity, and panel cells do not.

Funnel-plot and z-statistic diagnostics (Fig. 5 and *SI Appendix*, Table S4) show no strong corpus-wide publication-bias signature. PET- and PEESE-corrected pooled effects of 8.4% (95% CI 2.2 to 14.9) and 10.2% (95% CI 4.8 to 15.9) are similar in magnitude to the primary 9.9% pool, so the headline premium is not entirely an artifact of publication bias. Within the observational instrumental-variable cell, the largest estimates lie in the upper-right region of the funnel and the z-statistic distribution skews positive; in randomization, regression-discontinuity, and panel cells, neither pattern is visible.

## Discussion

This meta-analysis changes the interpretation of the “naturalization premium.” The positive average in the published literature is a real regularity in reported estimates, but it should not be treated as a design-invariant causal quantity. Across studies, and within the randomized trial itself, the estimated premium changes when the identifying comparison changes. This pattern points to selection into naturalization as a central part of the empirical object: Observational estimates partly describe who naturalizes and when, not only what citizenship does after naturalization.

This conclusion does not imply that citizenship has no economic value. Legal security, labor-market access, and signaling remain credible mechanisms, and some identification-credible estimates are positive. The evidence instead narrows what can be inferred from the conventional premium. It should be read as a design-bound summary of heterogeneous estimates, not as a portable forecast of the causal return to citizenship.

Nor is our conclusion the last word on the returns to citizenship. The analysis has limitations, and these point to priorities for future research. The earnings sample is modest and unevenly distributed across identification strategies: The randomization and regression-discontinuity cells contain one study each. These design cells are therefore also single-context cells, so their pooled estimates should be read as evidence from specific study settings rather than as fully general design averages. The near-zero randomized result is especially informative because its interval excludes premiums as large as the pooled average, but it should not be treated as a design average for all citizenship regimes, populations, or policy margins. The most valuable additions to this literature are therefore more of both: randomized encouragement designs built around interventions such as application fees, legal assistance, information, or administrative support, and regression-discontinuity designs that exploit sharp and clean institutional thresholds in citizenship access, where applicants cannot sort themselves across the cutoff.

The geography of the evidence is also narrow. Reflecting where the earnings literature currently exists, the included studies come mostly from Western and Northern Europe, Canada, and the United States; the resulting estimates therefore say less about citizenship effects in newer destination countries such as Poland, Colombia, Chile, Turkey, and South Korea, or in lower-income institutional contexts.

Our substantive and methodological findings of inflated estimates in observational studies of the returns to citizenship suggest three implications. For empirical research, identification strategy should be a first-order feature of how naturalization-premium estimates are summarized and cited. For policy, forecasts that citizenship reform will generate large earnings gains should be calibrated against identification-credible estimates rather than the observational average alone. Given the uncertainty that remains about the causal premium, governments considering costly naturalization programs or reforms premised on earnings gains may obtain a higher return by first investing in randomized evaluations of the economic returns to citizenship, which can be embedded in existing application-support programs at comparatively low cost. For the meta-analytic agenda, the binding constraint is not another correction to the same heterogeneous corpus; it is more plausibly unconfounded variation in citizenship access and uptake. Additional randomized encouragement designs and regression-discontinuity studies would do the most to sharpen the field’s answer. More panel studies alone are unlikely to settle it: Our within-study diagnostic shows that flat pretrends can coexist with sizable posttreatment selection bias, so new panel designs are informative mainly when naturalization timing is shifted by eligibility rules or policy changes rather than by immigrants’ own choices. Further observational instrumental-variable studies built on instruments of convenience would add the least: Without an instrument that is created by design, or close to it, they are more likely to contribute inflated estimates than evidence.

## Materials and Methods

### Search and Study Selection.

Starting from the published appendix list of naturalization-premium studies in Hainmueller et al. (13), we conducted forward-citation searches in OpenAlex, Semantic Scholar, and OpenCitations from 1975 onward. The seed list covered citizenship effects on immigrant outcomes broadly, not only earnings. Candidate records were screened on title and abstract using a structured controlled-vocabulary protocol, then on full text (*SI Appendix*, Fig. S1). We retained only studies estimating the effect or association of citizenship, naturalization, or access to citizenship on immigrant outcomes; we excluded reverse-direction studies of determinants of naturalization. For this earnings-focused analysis we further required an earnings, wage, salary, or income outcome and a reported or reconstructable uncertainty measure. Final published versions were used wherever available; working papers were retained only when no published version existed (5, 6, 7, 8, 9, 10, 11, 12, 13, 15, 19, 20, 21, 22, 23, 24, 25, 26, 27, 28, 29, 30, 31, 32, 33, 34, 35, 36, 37, 38, 39). The full search and screening protocol is in *SI Appendix*.

### Extraction and Coding.

Each study contributes one or more estimate-level rows for each substantively distinct outcome × subgroup × treatment estimand × specification combination. Each row records the raw effect, its sign convention, transformations applied to obtain a log-equivalent percent change, and the original-scale uncertainty. When a study reports multiple specifications for the same earnings contrast, the preferred pool uses the authors’ primary or preferred estimate; if no preferred estimate is named, we use the most complete main-text specification for the study’s central design and broadest eligible sample. When both reduced-form access/eligibility estimates and scaled actual-citizenship or LATE estimates are available from the same study, the primary pool uses the actual-citizenship or LATE contrast and reports the access/eligibility estimates separately as diagnostics. The moderator analyses use identification strategy plus six additional features: treatment estimand, effect measurement window, outcome data source, earnings-sample basis, destination country, and citizenship regime measured by the 2019 MIPEX Access to Nationality score.

### Effect Coding.

The primary metric is the log-equivalent percent earnings effect: For log earnings or log income coefficients, the reported coefficient is taken as the log effect and reported as 100[exp(β)−1]; base-10 log coefficients are converted to natural-log equivalents; level coefficients are converted only when the paper reports a percent effect or when a defensible baseline is available, with SEs propagated by the delta method. Sign conventions are normalized so that positive values indicate higher earnings under citizenship or citizenship access.

### Meta-Analytic Models and Diagnostics.

The primary specification is a multi level random-effects model with random intercepts for dataset family, study, and within-study sample. Eight dataset families pool studies that draw on the same underlying register or survey lineage—Statistics Norway linked registers, Statistics Sweden LINDA/register, Statistics Canada Census, INSEE administrative and tax records, Swiss Labour Force Survey, U.S. decennial Census, American Community Survey, and Current Population Survey—and the remaining studies enter as singleton families (*SI Appendix*, section S3). The model is fit by REML. Two-level, study-country-collapsed, and study-collapsed specifications serve as robustness checks; the two-level model uses study-clustered CR2 inference, and the cumulative design-tier diagnostic pools study-collapsed estimates as increasingly observational designs enter the corpus.

Heterogeneity is examined by stratifying the primary model on identification strategy and by fitting moderator meta-regressions for the main coded study features: identification strategy, citizenship regime, destination country, effect measurement window, outcome data source, earnings-sample basis, and treatment estimand. We summarize moderator models by their proportional reduction in between-estimate variance relative to the null model, and report conditional specifications that add identification strategy after the nonidentification moderators, with pooled destination-country fixed effects replacing the country-level MIPEX score in the country-adjusted specification. Moderator models are fit on the MIPEX-matched subset (55 estimates from 29 studies); full-sample sensitivity is reported in *SI Appendix*. The within-study Hainmueller et al. diagnostic compares randomized voucher assignment with citizenship uptake among experimental control-group immigrants who were not encouraged, using the Callaway–Sant’Anna difference-in-differences estimator in the balanced credit-bureau panel; *SI Appendix* reports two-way fixed-effects and fixed-effects counterfactual-imputation versions. Publication-bias diagnostics include funnel plots, FAT-PET and PEESE corrections (40), and stratified z-statistic distributions following Brodeur, Cook, and Heyes (41). Full definitions and model output are in *SI Appendix*.

### AI.

The authors used OpenAI Codex, based on GPT-5, and Anthropic Claude Code, based on Claude Opus 4.7 and Claude Fable 5, to assist with manuscript preparation, including text editing, consistency checks across the manuscript and *SI Appendix*, and updates to analysis code, tables, figures, and LaTeX files. The authors reviewed, revised, and fact-checked all AI-assisted output and are solely responsible for the final content. No AI-generated images or graphics are included.

## Supplementary Material

Appendix 01 (PDF)

## Data Availability

All data and code needed to reproduce the results in this article are deposited at Zenodo (DOI: 10.5281/zenodo.22141014). Person-level records underlying the within-study component of Hainmueller et al. (13) are restricted under a data-use agreement and are not included; the deposit contains the aggregated estimates and complete analysis code for that component.
