# Supplementary material for: The economic returns to citizenship: Evidence from a meta-analysis
Source: Proc Natl Acad Sci U S A. 2026 Sep 28;123(40):e2617804123. doi: 10.1073/pnas.2617804123 (PMC13643161; doi:10.1073/pnas.2617804123)
Supplement: Supplementary file 1 [file pnas.2617804123.sapp.pdf]

Supporting Information for "The Economic Returns to Citizenship: Evidence from a Meta-Analysis"

Jens Hainmueller and David D. Laitin

Correspondence: David D. Laitin, dlaitin@stanford.edu

Contents

S1 Search and study selection **1**

S2 Extraction and coding **3**

S3 Statistical model **6**

S4 Supplementary tables **11**

S5 Data availability **14**

S1. Search and study selection

Seed corpus

The starting point for the literature search was the appendix list of studies analyzing the naturalization premium published as Table S25 in the supporting information of Hainmueller et al. (1). That list was constructed by Hainmueller et al. (1) as a cited-paper and snowball bibliography of the prior citizenship-premium literature: they began with highly cited Google Scholar papers that cited the seminal studies by Chiswick (2) or Bratsberg et al. (3) and whose abstracts advertised a statistical test of the returns to citizenship, then expanded the list through references and keyword-based snowballing around immigration and citizenship. The seed list was not restricted to earnings: it included studies of citizenship effects on labor-market, education, health, social-integration, demographic, and other immigrant outcomes. These 53 seed studies span 1978–2024 and cover the major empirical traditions in the citizenship-effects literature: cross-sectional naturalization-status comparisons, panel fixed-effects analyses, policy-reform difference-in-differences, regression discontinuity designs, and observational instrumental variables. The randomized fee-voucher encouragement design in Hainmueller et al. (1) entered the corpus through the forward-citation and full-text update rather than through the seed list itself.

We treated this seed list as the entry point but did not accept it wholesale. Eleven seed studies were excluded during full-text review: five as review or synthesis articles rather than primary effect-estimation studies (4–8); three for lacking any numeric standard errors (9–11); two for treating naturalization as an outcome or providing descriptive profiles rather than estimating citizenship effects (12, 13); and one book chapter (14) that summarizes two analyses already included as journal

articles (Steinhardt (32), Germany; Steinhardt and Wedemeier (31), Switzerland). Forty-two seed studies survive into the active corpus alongside the forward-citation expansion.

Forward citation search

For each seed study we conducted forward-citation searches in OpenAlex, Semantic Scholar, and OpenCitations COCI, restricted to publication year 1975 or later. Seed studies were matched to citation databases by DOI when available and otherwise by title, author surname, and publication year. The combined search returned 12,285 citing records, which were deduplicated by DOI when available and otherwise by normalized title, first-author surname, and year, yielding 5,116 unique candidate records.

Title and abstract screening

The 5,116 candidates were screened by title and abstract. Inclusion required mention of at least one citizenship/naturalization term, at least one immigrant-population term, and at least one outcome or methodology term. Studies about determinants of naturalization (e.g., interest in citizenship, intentions to naturalize, naturalization rates, or take-up as the dependent variable) and normative or theoretical analyses without empirical estimation were excluded. The screening produced 436 candidates for full-text review.

Additional screening

The 436 first-pass includes were reviewed against the inclusion criteria. This pass retained 45 records for full-text review, sent 44 to manual review, and excluded 347 records, mostly generic immigrant-outcome studies without a citizenship-as-treatment specification or seed-corpus duplicates. A second check of the unclear or excluded candidates identified 7 false-negative includes plus 8 additional records for manual review.

Full-text review

One author adjudicated the 52 records marked for manual review, with 24 included and 28 excluded. Final published versions were used whenever available; working papers were retained only when no published version existed. When supplementary appendices or online supporting materials were available, they were included with the main article for full-text review and extraction. Of the 76 records retained for full-text review (45 from the first screening pass, 7 recovered in the second check, and 24 from manual review), 36 were excluded during full-text review and extraction: duplicate or superseded versions of studies already in the corpus, records whose full text could not be retrieved, studies outside the target scope on full-text reading, studies for which no usable estimate or uncertainty measure could be coded, and unpublished theses or preprints without an eligible published version. One additional eligible study identified from reference lists during full-text review was added to the corpus, leaving 83 studies in the eligible corpus (42 surviving seed studies plus 41 additional studies).

Earnings restriction

For this earnings-focused meta-analysis we further required that each included study report at least one earnings, wage, salary, or income outcome with a reported or reconstructable uncertainty measure. The same usable-uncertainty requirement applied at full-text review; Riphahn and Saif (15),

for example, was excluded at that stage because the relevant tables report point estimates without standard errors, confidence intervals, or other information sufficient to reconstruct uncertainty. Studies measuring only employment, education, health, social integration, fertility, or other non-earnings outcomes were excluded from the pooled earnings analysis. The earnings sample contains 60 preferred estimates from 31 studies. The full PRISMA-style flow is shown in Figure S1.

S2. Extraction and coding

Coding procedure

For each eligible study, we coded one study-level record and one or more estimate-level records. Estimate rows were defined by substantively distinct combinations of outcome, subgroup, country, treatment estimand, and specification. Each row records the original point estimate and uncertainty measure, the reported model or table source, the treatment contrast, the outcome definition, the identification strategy, whether the contrast targets actual citizenship or access/eligibility, the outcome timing and data source, and the transformations needed to place earnings effects on a common log-equivalent percent scale. The preferred earnings pool was then restricted to rows marked ready for meta-analysis, not requiring human verification, and flagged as preferred. For duplicate specifications of the same earnings contrast, the preferred flag follows the authors' primary or preferred estimate; if no preferred estimate was named, we used the most complete main-text specification for the study's central design and broadest eligible sample. Distinct estimands, such as randomized access/encouragement and citizenship uptake, were coded separately at extraction. For the primary estimand-harmonized pool, when both reduced-form access/eligibility estimates and scaled actual-citizenship or LATE estimates were available from the same study, we retained the actual-citizenship or LATE contrast and kept the access/eligibility rows for diagnostic reporting. The Hainmueller et al. voucher-assignment ITTs and voucher-induced citizenship LATEs are therefore shown together in Table S6, but only the LATE rows enter the primary randomized cell. Selected-status diagnostics from the Hainmueller et al. trial are reported only as within-study selection checks and are excluded from the preferred meta-analytic pool. Coding checks verified required fields, controlled vocabularies, sign conventions, log-equivalent conversions, dependency identifiers, and consistency between point estimates and reported uncertainty.

Coded fields and extracted moderators

For each study and estimate, we coded the features below as moderators in the heterogeneity analyses. The focal moderator is identification strategy: a single categorical variable recording the source of identifying variation.

Extracted moderators.

Moderator	Coding
Identification strategy	Randomization, regression discontinuity, policy or cohort reform, panel within-unit, observational instrumental variables, or cross-sectional observational.

Continued on next page

Moderator	Coding
Treatment estimand	Actual citizenship/naturalization or status contrasts, LATEs for citizenship uptake, and reduced-form access/eligibility contrasts through policy exposure or randomized encouragement. When a study reports both reduced-form access and scaled actual-citizenship/LATE contrasts, the primary pool uses the actual-citizenship/LATE contrast and reports the reduced-form rows separately.
Effect measurement window	Time between the relevant treatment or exposure and the earnings outcome. For the moderator analyses, we code this as a categorical factor with four main categories: contemporaneous, short-term follow-up ($>$ 0–2 years), medium-term follow-up ($>$ 2–5 years), and long-term follow-up ($>$ 5 years). Cross-sectional citizenship-status comparisons enter the contemporaneous category when citizenship status and earnings are measured at the same time; short-term is reserved for post-treatment outcomes.
Outcome data source	Two-category indicator for administrative versus self-reported earnings data. Administrative data include linked tax, payroll, credit, and register records; census and survey earnings are coded as self-reported.
Earnings-sample basis	Two-category indicator for whether the estimate uses a broad income sample that includes zero earners or household income, or restricts to employed workers or positive earners.
Destination country	Destination country for the estimate. Most studies contribute estimates for a single destination country; in multi-country studies, destination country is coded at the estimate level. One pooled cross-country estimate is coded as multi-country.
Citizenship regime	2019 MIPEX Access to Nationality score (16), a country-level index of how inclusive or restrictive access to citizenship is. Higher scores indicate more inclusive access.

Outcome harmonization

The primary effect-size metric is a log-equivalent percent earnings effect computed as $100[\exp(\beta)-1]$ for log-earnings or log-income coefficients. Base-10 log coefficients are converted to natural-log equivalents by dividing by $\log_{10}(e)$. Level coefficients are converted to log-equivalent percent only when the paper reports a percent effect or when a defensible baseline mean is available; otherwise the row is excluded from the pooled earnings analysis. Standard errors for converted percent effects are obtained by the delta method.

Sign convention

All effects are coded so that a positive sign means citizenship, naturalization, or access to citizenship increases earnings relative to the comparison group. When a paper's reported coefficient uses the reverse contrast (e.g., longer waiting period as the regressor), the sign is reversed before applying transformations and the sign flip is documented per row.

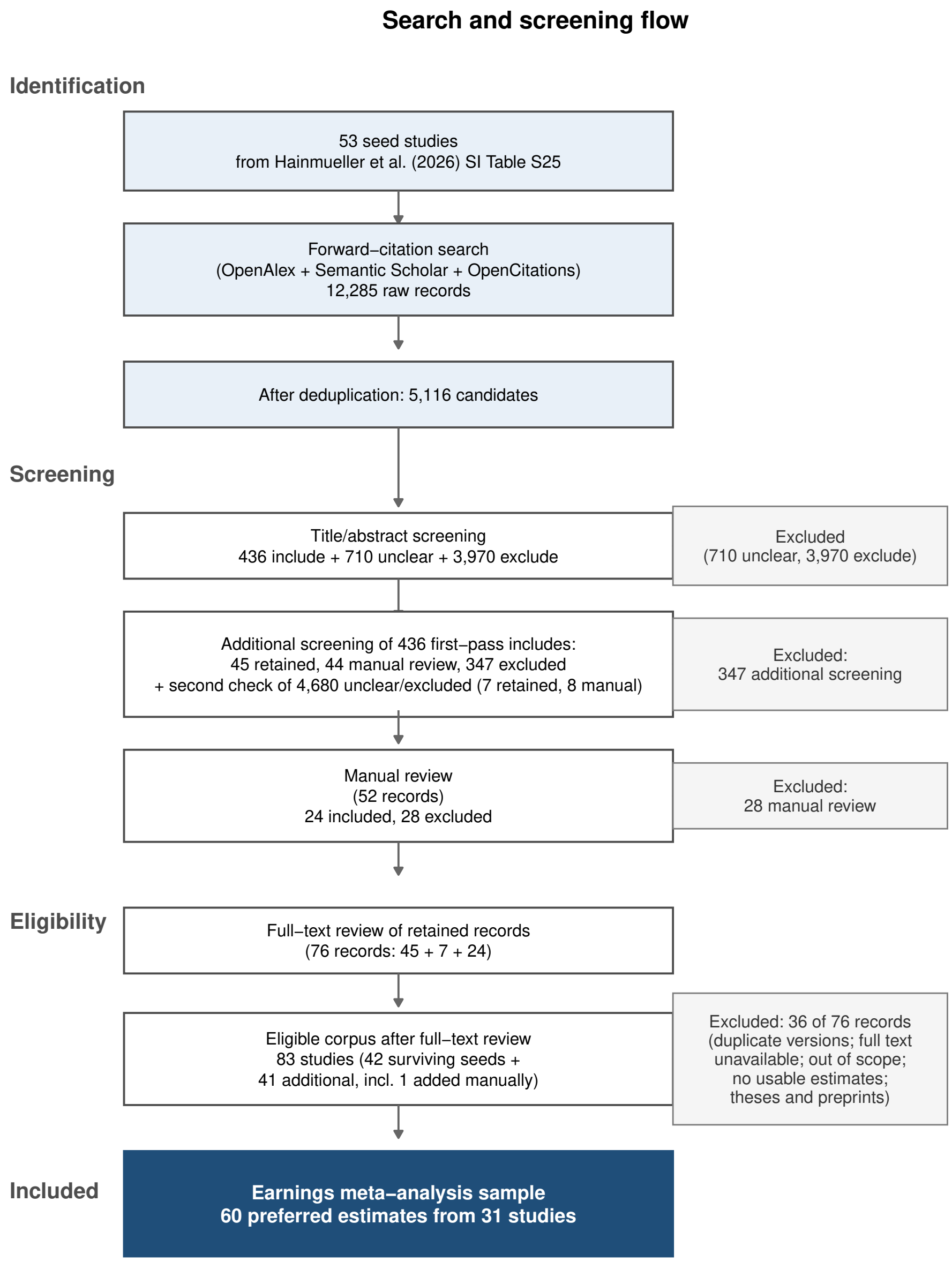

Figure S1: **Search and screening flow.** The diagram traces the corpus from the 53 seed studies through forward-citation expansion, deduplication, title and abstract screening, full-text review, and the earnings restriction.

S3. Statistical model

Primary specification

The primary pooled estimate uses a multilevel random-effects meta-analysis (`rma.mv` from the R package `metafor`) on log-equivalent percent earnings effects:

$$y_{dsi} = \mu + u_d + u_s + u_{si} + \varepsilon_{dsi},$$

where y_{dsi} is the ith estimate in study s from dataset family d. Dataset families group studies that draw on the same underlying register or survey lineage. The term $u_d \sim N(0, \tau_d^2)$ is a dataset-family random intercept, $u_s \sim N(0, \tau_s^2)$ is a study random intercept, u_{si} captures within-study sample dependence, and ε_{dsi} has variance $\sigma_{dsi}^2 = \mathrm{SE}_{dsi}^2$. Variance components are estimated by REML. The three-level model uses Wald inference; the two-level robustness model with study and within-study sample random effects uses study-clustered CR2 inference (`coef_test` from `clubSandwich`).

We code eight dataset families based on the data lineage reported in each study; remaining studies enter as singleton families.

- **Norway register** (Statistics Norway FD-Trygd / linked administrative registers): Hayfron (25), Bratsberg and Raaum (29).
- **Sweden register** (Statistics Sweden linked register databases, including LINDA and STATIV): Engdahl (30), Engdahl (34), and the Swedish arm of Pendakur and Bevelander (35).
- **Canada Census** (Statistics Canada Census public-use and long-form microdata): the Canadian arm of Chiswick and Miller (23), Pivnenko and DeVoretz (21), DeVoretz and Pivnenko (22), and the Canadian arm of Pendakur and Bevelander (35).
- **France administrative** (INSEE Échantillon Démographique Permanent with linked DADS earnings records): Jarreau (44), Govind (47).
- **Switzerland LFS** (Swiss Labour Force Survey, SAKE): Steinhardt and Wedemeier (31, Switzerland), Pecoraro and Wanner (40).
- **U.S. Census** (U.S. decennial Census public-use microdata): Chiswick (2), the U.S. arm of Chiswick and Miller (23), Akbari (24), Mazzolari (27).
- **U.S. ACS** (American Community Survey IPUMS microdata): Zhou and Lee (33), Chi and Coon (43).
- **U.S. CPS** (Current Population Survey March supplement): Shierholz (28), Kim et al. (36).

Other studies—including Steinhardt (32, Germany; IAB register), Gathmann and Keller (39; German Microcensus), Sargsyan (37; Luxembourg Income Study), Zanoni and He (48; Canadian Labour Force Survey), Bratsberg, Ragan, and Nasir (3; mixed NLSY/Census/CPS), Crown and Faggian (41; NSF Survey of Doctoral Recipients), Hainmueller et al. (42; municipal referendum records linked to Swiss OASI social security earnings records), Hainmueller et al. (1; voucher-lottery and credit-bureau records), Peters et al. (46; Netherlands register), Gritti et al. (49; Italian linked data), Soon (38; 2008 European Values Study, multi-country), and Kahanec and Zaiceva (26; EU-SILC 2005, EU15/EU8 aggregates)—enter as singleton families because they draw on data lineages not shared with another study in the corpus.

Estimated variance components for the primary model are $\tau_d^2 = 0.0000$ at the dataset-family level, $\tau_s^2 = 0.0111$ at the study level, and $\tau_{si}^2 = 0.0044$ within studies (total $\tau^2 = 0.0155$). We report $I^2 = 99.5\%$ using the generalized Higgins–Thompson formula $I^2 = \tau^2/(\tau^2 + v_{\text{typical}})$, with

$v_{\text{typical}} = (k-1)\sum w_i/[(\sum w_i)^2 - \sum w_i^2]$ and $w_i = 1/\text{vi}$. The dataset-family component is small because after coding eight families explicitly, the residual covariance among studies in the same family is largely absorbed by the study-level random intercept; we retain the level for transparency.

Robustness specifications

Two-level (study + within-study sample), study-collapsed, and study-country-collapsed specifications are reported alongside the primary model. The primary row-level model uses all 60 estimand-harmonized preferred estimates from 31 studies. The study-collapsed specification uses one inverse-variance summary per study (31 observations). The study-country-collapsed specification uses one inverse-variance summary per study-destination-country pair (35 observations), because Chiswick and Miller (23), Kahanec and Zaiceva (26), Pendakur and Bevelander (35), and Sargsyan (37) each contribute two destination-country or regional cells.

Stratified pooling

Per-identification-strategy pooled estimates are computed with the same multilevel specification on the corresponding subsample where the cluster structure permits. For each multilevel subgroup fit, confidence intervals use cluster-robust (CR2) standard errors clustered by dataset family when that adjustment is estimable (the policy/reform and panel cells); when the random-effects structure is not nested within the clustering variable (the cross-sectional and observational-IV cells), Wald intervals from the multilevel model are reported instead. The randomized and regression-discontinuity cells each contain two estimates from a single study, so the multilevel clustered specification is not estimable; these cells are pooled with a univariate REML model and Wald inference instead. The aggregated credibility-tier pools reported in the main text follow the same rule: the most-credible (RCT + RDD + policy/cohort reform) and panel blocks report dataset-family CR2 intervals, and the observational block (cross-sectional + observational IV) reports multilevel Wald intervals because the non-nested random effects preclude the CR2 adjustment.

Alternative-moderator robustness check

To test whether the identification-strategy gradient is confounded by other study features, we fit multilevel meta-regressions using the preferred row-level earnings estimates and the moderator fields defined above. Citizenship regime is measured with the continuous 2019 MIPEX Access to Nationality score from the Migrant Integration Policy Index 2020 (16). Destination country enters as fixed effects, with sparse countries pooled for estimation. These moderator-comparison models are fit by maximum likelihood (ML) rather than REML so that the variance components and likelihood-based information criteria are comparable across specifications with different fixed-effect terms; the primary pooled model in Section S3 uses REML.

Table S5 reports each moderator one at a time and then two conditional models. The first adds identification strategy after citizenship regime, effect measurement window, outcome data source, earnings-sample basis, and treatment estimand. The second repeats this calculation after replacing the country-level MIPEX score with pooled destination-country fixed effects. The country-inclusive specification is parameter-heavy (15 and 19 model parameters before and after adding identification strategy, respectively, on 55 estimates), but it converges and yields a similar conclusion: adding identification strategy reduces residual between-estimate variance by partial $R^2_\tau = 0.797$. We summarize explanatory power as the proportional reduction in total estimated between-estimate variance, R^2_τ, relative to the null multilevel model. For the adjusted rows, partial R^2_τ is the

proportional reduction in residual between-estimate variance from adding identification strategy after the corresponding non-identification baseline.

The MIPEX-matched sample contains 55 estimates from 29 studies because two studies report estimates for multi-country aggregates with no 2019 MIPEX score: Kahanec and Zaiceva (26), which reports separate EU15 and EU8 estimates, and Soon (38), which pools multiple European host countries. As a sensitivity check, we refit the moderator horse race on the full primary sample of 60 estimates from 31 studies, omitting the MIPEX moderator (whose merge requires a single destination country). Identification strategy still dominates: $R^2_\tau = 0.693$ on its own and a partial $R^2_\tau = 0.706$ after the remaining non-identification moderators (effect measurement window, outcome data source, earnings-sample basis, and treatment estimand). The relative ranking of moderators is unchanged.

Within-study selection diagnostic

For Hainmueller et al. (1), the voucher-induced citizenship LATE rows are the randomized preferred rows in the primary meta-analysis; the voucher-assignment ITTs are retained in the all-estimands audit and reported in Table S6 rather than averaged with the LATEs. To show how selection can re-enter when the experimental design is ignored, the main text applies the Callaway–Sant'Anna difference-in-differences estimator for staggered treatment timing (17). Figure S2 reports the corresponding two-way fixed-effects event-time specification using the authors' replication data and code. Figure S3 repeats the side-by-side comparison using the fixed-effects counterfactual-imputation estimator of Liu et al. (18). This estimator fits a two-way fixed-effects outcome model using untreated observations, imputes untreated potential outcomes for treated observations, and averages observed-minus-imputed gaps by event time. The main-text figure and both event-time figures restrict the matched six-year credit-bureau panel to individuals with observed income in all six panel years. Results are substantively similar across the three estimators: the randomized voucher path remains close to zero, while the selected citizenship path rises after treatment. In both SI figures, the left panel estimates randomized voucher effects and the right panel estimates effects of citizenship uptake among experimental control-group immigrants who were not encouraged. Table S6 reports the experimental income estimates alongside observational panel diagnostics from the same credit-bureau panel. The selected-status rows are shown only as the within-study diagnostic.

Publication-bias diagnostics

FAT-PET and PEESE corrections (19) are estimated as random-effects (REML) meta-regressions on the study-country-collapsed dataset (`rma.uni` in `metafor`). The FAT-PET regression is $\text{effect}_s = \beta_0 + \beta_1 \text{SE}_s + \varepsilon_s$ and PEESE is $\text{effect}_s = \beta_0 + \beta_1 \text{SE}_s^2 + \varepsilon_s$, with the publication-bias-corrected pooled estimate given by β_0; weights are the inverse of the sampling variance plus the estimated residual heterogeneity. Funnel plots and z-statistic distributions stratified by identification strategy follow Brodeur et al. (20).

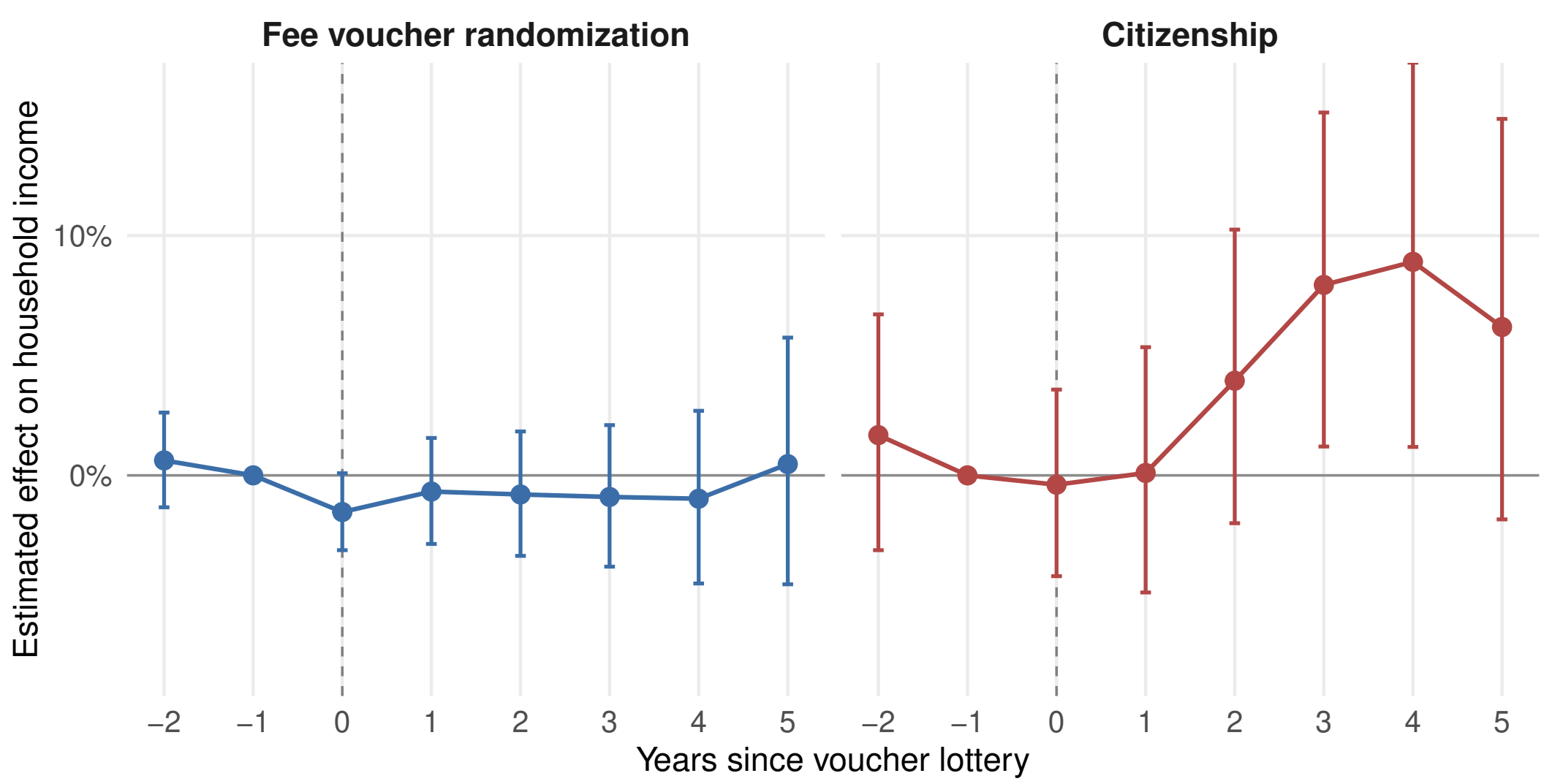

Figure S2: **Two-way fixed-effects event-study version of the within-study selection check in Hainmueller et al. (1).** Unlike the main-text figure, which uses the Callaway–Sant'Anna estimator, this figure estimates a conventional two-way fixed-effects event-study regression—the specification most panel studies in the corpus use. The left panel estimates voucher-assignment effects in the matched six-year credit-bureau panel, restricted to individuals with observed income in all six panel years, using individual and calendar-year fixed effects, the original randomization-block weights, and standard errors clustered by individual. The right panel estimates effects of citizenship uptake among experimental control-group immigrants who were not encouraged. The omitted reference period is one year before the lottery; the vertical dashed line marks the lottery year. Coefficients are transformed to percent effects using $100\{\exp(\hat{\beta}) - 1\}$. The right panel is a selection diagnostic, not a randomized estimate.

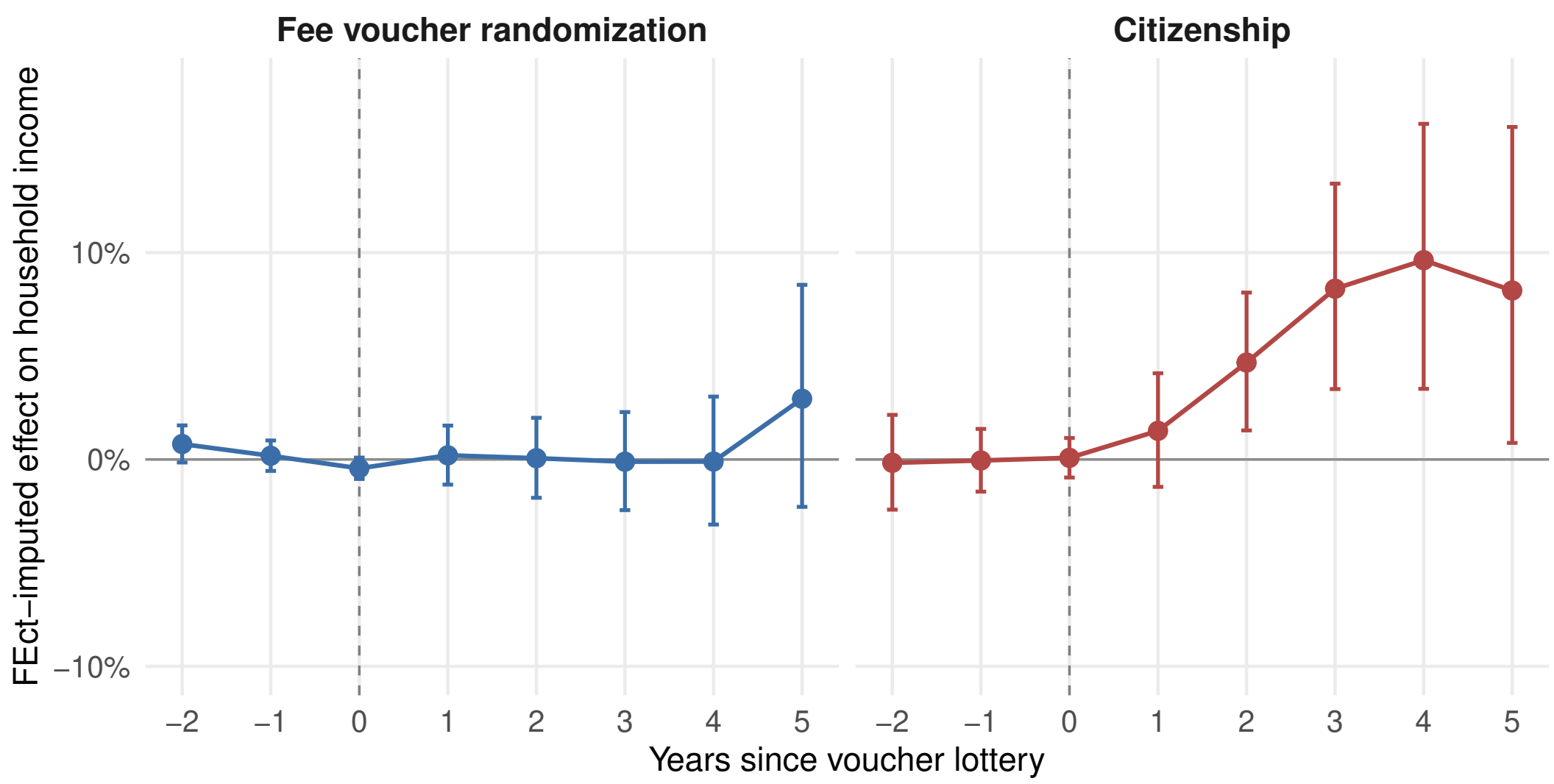

Figure S3: **Fixed-effects counterfactual-imputation estimates in Hainmueller et al. (1).** Both panels use the matched six-year credit-bureau panel, restricted to individuals with observed income in all six panel years, and the fixed-effects counterfactual-imputation estimator of Liu et al. (18), implemented in the R package `fect`. The left panel estimates randomized voucher-assignment effects; the right panel estimates effects of citizenship uptake among experimental control-group immigrants who were not encouraged. Coefficients are transformed to percent effects using $100\{\exp(\hat{\beta}) - 1\}$. Unlike the two-way fixed-effects specification in Figure S2, the fect estimates do not apply the randomization-block weights (the installed `fect` interface accepts no weight argument); confidence intervals are from the package's nonparametric bootstrap, which resamples individuals and therefore clusters uncertainty at the individual level. The pattern is similar to the Callaway–Sant'Anna estimates in the main text: the randomized voucher path remains close to zero, while the selected citizenship path rises after treatment.

S4. Supplementary tables

Table S1: Studies contributing preferred earnings estimates

Study	Country	Design	Estimand	Outcome source	Earnings sample	Provenance	k	Effect [95% CI]
Chiswick 1978	United States	Cross-sectional obs.	status association	census	positive earners only	Footnote 26, regression 2 (OLS)	1	7.0 [-2.2, 17.1]
Chiswick and Miller 1992	Canada; United States	Cross-sectional obs.	status association	census	employed workers only	Table 7-8, column b (OLS)	2	5.1 [3.2, 7.0]
Bratsberg et al. 2002	United States	Panel within-unit	status association	survey self report	employed workers only	Table 5, column 1 (FE)	1	5.7 [1.7, 9.9]
Pivnenko and DeVoretz 2003	Canada	Cross-sectional obs.	status association	census	positive earners only	Table 4 (OLS)	1	17.2 [15.6, 18.9]
DeVoretz and Pivnenko 2005	Canada	Cross-sectional obs.	status association	census	positive earners only	Table 3, column 1 (OLS)	4	7.9 [6.6, 9.3]
Akbari 2008	United States	Cross-sectional obs.	status association	census	employed workers only	Table 3, column 1 (OLS)	4	8.3 [7.8, 8.8]
Hayfron 2008	Norway	Cross-sectional obs.	status association	administrative register	employed workers only	Table 3, column 6; text calculation below Table 3 (RE)	1	10.2 [2.2, 18.8]
Kahanec and Zaiceva 2009	EU15; EU8	Cross-sectional obs.	status association	survey self report	employed workers only	Table IV, column 3 (OLS)	4	3.9 [2.0, 5.8]
Mazzolari 2009	United States	Policy/reform	itt access	census	positive earners only	Table 6, Panel A (DiD)	1	1.2 [-0.8, 3.2]
Shierholz 2010	United States	Cross-sectional obs.	status association	survey self report	household income all respondents	Table 3, column 3 (OLS)	1	15.7 [10.5, 21.2]
Bratsberg and Raaum 2011	Norway	Panel within-unit	status association	administrative register	employed workers only	Table 7.4, Model III, All (FE)	2	-1.6 [-2.3, -0.9]
Engdahl 2011	Sweden	Panel within-unit	status association	administrative register	positive earners only	Table 3.A2.8 (panel)	2	-2.4 [-4.2, -0.5]
Steinhardt 2012	Germany	Panel within-unit	status association	administrative register	employed workers only	Table 4 (FE)	2	0.6 [-0.1, 1.3]
Steinhardt and Wedemeier 2012	Switzerland	Cross-sectional obs.	status association	survey self report	employed workers only	Table 2 (decomposition)	3	11.5 [3.8, 19.8]
Zhou and Lee 2013	United States	Cross-sectional obs.	status association	census	employed workers only	Table 3 (OLS)	1	33.6 [-9.7, 97.8]
Engdahl 2014	Sweden	Panel within-unit	status association	administrative register	positive earners only	Table 4, Model III (FE)	2	3.8 [3.1, 4.6]
Pendakur and Bevelander 2014	Canada; Sweden	Observational IV	late citizenship	administrative register; census	positive earners only	Table 3 (IV/2SLS)	4	70.2 [65.7, 74.9]
Kim et al. 2015	United States	Observational IV	ate citizenship	survey self report	household income all respondents	Table 4, earnings equation (Treatment-effect model using family income...)	1	31.3 [23.0, 40.0]
Sargsyan 2017	Denmark; France	Cross-sectional obs.	status association	survey self report	employed workers only	Table 4 (decomposition)	4	1.8 [-3.3, 7.3]
Soon 2017	Multiple European host countries	Observational IV	late citizenship	survey self report	household income all respondents	Table V, Specification 1 (IV/2SLS)	1	53.0 [4.8, 123.3]
Gathmann and Keller 2018	Germany	Policy/reform	eligibility or policy effect	census	employed workers only	Table 3, column 4 (Reduced-form eligibility regression, Table...)	2	0.8 [0.4, 1.3]
Pecoraro and Wanner 2018	Switzerland	Policy/reform	eligibility or policy effect	survey self report	employed workers only	Table 1 (DiD)	1	-0.6 [-12.0, 12.2]
Crown and Faggian 2019	United States	Panel within-unit	ate citizenship	survey self report	employed workers only	Table 4, column 1 (OLS)	1	9.9 [4.4, 15.6]
Hainmueller et al. 2019	Switzerland	RDD	itt access	administrative register	includes zero earnings	Supplementary Table S5, column 6; Figure 1D and Results text (DiD)	2	11.3 [5.2, 17.8]

Continued on next page

Table S1 continued from previous page

Study	Country	Design	Estimand	Outcome source	Earnings sample	Provenance	k	Effect [95% CI]
Chi and Coon 2020	United States	Cross-sectional obs.	status association	census	positive earners only	Table 3, column 1 (OLS)	1	7.5 [6.6, 8.4]
Jarreau 2020	France	Observational IV	late citizenship	tax record	employed workers only	Table 9, column 3 (IV/2SLS)	1	24.6 [-26.6, 111.5]
Peters et al. 2020	Netherlands	Panel within-unit	status association	administrative register	employed workers only; includes zero earnings	Table 1, Model 1 (FE)	4	2.6 [1.9, 3.2]
Govind 2021	France	Policy/reform	itt access	administrative register	employed workers only	Table 3 (DiD)	2	8.9 [2.9, 15.3]
Zanoni and He 2021	Canada	Policy/reform	itt access	survey self report	employed workers only	Table 5, Column 1, Policy Implementation (DiD)	1	14.1 [7.0, 21.7]
Gritti et al. 2025	Italy	Cross-sectional obs.	ate citizenship	linked data	positive earners only	Figure 6, full sample, 2014, + Sociodemo t-1 (OLS)	1	7.6 [-28.1, 61.1]
Hainmueller et al. 2026	United States	Randomization/RCT	late citizenship	credit record	household income all respondents	Table 3, Panel B; SI Appendix Table S4, Panel B (IV/2SLS)	2	1.9 [-1.3, 5.3]

Notes: The provenance column identifies a representative preferred source for the study-level row and does not itemize subgroup-specific rows. Short model tags are OLS, FE (fixed effects), RE (random effects), DiD, RDD, ITT, IV/2SLS, panel, and decomposition. When $k > 1$, multiple preferred rows from the same study are collapsed into one study-level entry.

Table S2. Pooled effects by identification strategy

Design	k	Studies	Effect [95% CI]	p
Randomization/RCT	2	1	1.9 [-1.3, 5.3]	0.246
RDD	2	1	11.3 [5.2, 17.8]	<0.001
Policy/reform	7	5	4.7 [-2.8, 12.7]	0.155
Panel within-unit	14	7	4.2 [-2.6, 11.4]	0.173
Observational IV	7	4	53.0 [27.6, 83.5]	<0.001
Cross-sectional obs.	28	13	7.6 [5.0, 10.3]	<0.001

Table S3. Sign and significance by identification strategy

Design	Positive p<0.05	Positive n.s.	Negative p<0.05	Negative n.s.
Randomization/RCT	0	2	0	0
RDD	2	0	0	0
Policy/reform	4	2	0	1
Panel within-unit	8	2	2	2
Observational IV	6	1	0	0
Cross-sectional obs.	18	7	0	3

Table S4. PET and PEESE diagnostics

Diagnostic	k	Effect [95% CI]	p
PET	35	8.4 [2.2, 14.9]	0.007
PEESE	35	10.2 [4.8, 15.9]	<0.001

Notes: PET and PEESE are study-country-collapsed diagnostics; interpret cautiously because effects are heterogeneous.

Table S5. Moderator contribution to explained heterogeneity

Model	k	Studies	Parameters	R^2_τ	Partial R^2_τ
No moderators	55	29	1	0.000	
Identification strategy	55	29	6	0.684	
Citizenship regime (MIPEX)	55	29	2	0.044	
Destination-country fixed effects	55	29	9	0.111	
Effect measurement window	55	29	4	0.129	
Outcome data source	55	29	2	-0.019	
Earnings-sample basis	55	29	2	0.019	
Treatment estimand	55	29	2	0.015	
Identification strategy added after other moderators	55	29	12	0.800	0.704
Identification strategy added after other moderators plus country FE	55	29	19	0.894	0.797

Notes: Models use the row-level preferred earnings estimates matched to the 2019 MIPEX Access to Nationality score. R^2_τ is the proportional reduction in total estimated between-estimate variance relative to the null multilevel model. The first seven non-null rows show each reported moderator one at a time: identification strategy, citizenship regime (MIPEX Access to Nationality), destination country, effect measurement window, outcome data source, earnings-sample basis (broad income sample versus employed/positive earners), and treatment estimand. The first adjusted row adds identification strategy after the non-identification moderators: citizenship regime, outcome data source, earnings-sample basis, effect measurement window, and treatment estimand. Its partial R^2_τ is the proportional reduction in residual between-estimate variance relative to that omitted non-identification baseline. The final row repeats this calculation after replacing the country-level MIPEX score with pooled destination-country fixed effects.

Destination-country fixed effects pool countries with fewer than three row-level estimates into an other-country category.

Table S6. Within-study diagnostic in Hainmueller et al. (1)

Identification source	Estimated contrast	Sample and model	Effect [95% CI]	p
Voucher randomization	ITT: voucher access	Three-year credit-bureau income	0.0 [-2.3, 2.4]	1.000
Voucher randomization	LATE: citizenship uptake	Three-year credit-bureau income	2.5 [-5.4, 11.1]	0.542
Voucher randomization	ITT: voucher access	Six-year panel with individual and year fixed effects	0.2 [-1.6, 2.0]	0.824
Voucher randomization	LATE: citizenship uptake	Six-year panel with individual and year fixed effects	1.8 [-1.7, 5.5]	0.317
Selected application timing	Static panel: submitted N-400 vs. not	Control group only; individual and year fixed effects	3.7 [0.1, 7.4]	0.043
Selected application timing	Dynamic panel: effect after 4 years	Control group only; individual and year fixed effects	6.8 [1.5, 12.4]	0.011
Selected application timing	Dynamic panel: effect after 4 years	Full sample; individual and year fixed effects	6.1 [2.3, 10.0]	0.001

Notes: Outcome is log household income from the credit-bureau panel, reported as percent changes using $100\{\exp(\hat{\beta})-1\}$. Experimental rows use the voucher lottery or voucher-induced citizenship uptake. Observational rows ignore the randomized assignment and use selected N-400 submission timing; they are coded as selection diagnostics and excluded from the preferred meta-analytic pool.

S5. Data availability

All data and code needed to reproduce the results in this article are deposited at Zenodo (DOI: 10.5281/zenodo.22141014). The deposit contains the harmonized meta-analytic dataset (60 preferred estimates from 31 studies), all analysis code, and derived aggregate outputs. Restricted NaturalizeNY source data for Hainmueller et al. (1) cannot be shared under the relevant data-use agreement; analysis code and derived aggregate outputs are included. MIPEX Access to Nationality scores (16) are available from www.mipex.eu.

References

[1] Jens Hainmueller, Elisa Cascardi, Michael Hotard, Rey Koslowski, Duncan Lawrence, Vasil Yasenov, and David D. Laitin. The socioeconomic returns to citizenship: A randomized controlled trial. *Proceedings of the National Academy of Sciences*, 123(5):e2516178123, 2026. doi: 10.1073/pnas.2516178123.

[2] Barry R. Chiswick. The effect of Americanization on the earnings of foreign-born men. *Journal of Political Economy*, 86 (5):897–921, 1978. doi: 10.1086/260717.

[3] Bernt Bratsberg, James F. Ragan, and Zafar M. Nasir. The effect of naturalization on wage growth: A panel study of young male immigrants. *Journal of Labor Economics*, 20(3):568–597, 2002. doi: 10.1086/339616.

[4] Thomas Liebig. Main findings of the joint EC/OECD seminar on naturalisation and the socio-economic integration of immigrants and their children. In *Naturalisation: A Passport for the Better Integration of Immigrants?*, pages 15–20. OECD Publishing, Paris, 2011. doi: 10.1787/9789264099104-3-en.

[5] Madeleine Sumption and Sarah Flamm. The economic value of citizenship for immigrants in the United States. Report, Migration Policy Institute, National Center on Immigrant Integration Policy, September 2012.

[6] National Academies of Sciences, Engineering, and Medicine. *The Integration of Immigrants into American Society*. The National Academies Press, Washington, DC, 2015. doi: 10.17226/21746. Edited by Mary C. Waters and Marisa Gerstein Pineau.

[7] Christina Gathmann and Ole Monscheuer. Naturalization and citizenship: Who benefits? *IZA World of Labor*, 2020(125): 125v2, 2020. doi: 10.15185/izawol.125.v2.

[8] Christina Gathmann and Julio Garbers. Citizenship and integration. *Labour Economics*, 82:102343, 2023. doi: 10.1016/j.labeco.2023.102343.

[9] Pieter Bevelander and Justus Veenman. Naturalization and employment integration of Turkish and Moroccan immigrants in the Netherlands. *Journal of International Migration and Integration*, 7(3):327–349, 2006. doi: 10.1007/s12134-006-1016-y.

[10] Kirk Scott. The economics of citizenship: Is there a naturalization effect? In Pieter Bevelander and Don J. DeVoretz, editors, *The Economics of Citizenship*, pages 107–126. Malmö University (MIM), Malmö, 2008.

[11] Maria E. Enchautegui and Linda Giannarelli. The economic impact of naturalization on immigrants and cities. Research report, Urban Institute, December 2015.

[12] Fernando Mata. Patterns of acquiring citizenship. In Shiva S. Halli and Leo Driedger, editors, *Immigrant Canada: Demographic, Economic and Social Challenges*, pages 163–182. University of Toronto Press, Toronto, 1999. doi: 10.3138/9781442676022-010.

[13] Klaus F. Zimmermann, Amelie F. Constant, and Liliya Gataullina. Naturalization proclivities, ethnicity and integration. *International Journal of Manpower*, 30(1-2):70–82, 2009. doi: 10.1108/01437720910948401.

[14] Max Friedrich Steinhardt. The impact of naturalisation on immigrant labour market integration in Germany and Switzerland. In *Naturalisation: A Passport for the Better Integration of Immigrants?*, pages 145–151. OECD Publishing, Paris, 2011. doi: 10.1787/9789264099104-8-en.

[15] Regina T. Riphahn and Salwan Saif. Naturalization and labor market performance of immigrants in Germany. *Labour*, 33 (1):48–76, 2019. doi: 10.1111/labr.12136.

[16] Giacomo Solano and Thomas Huddleston. Migrant integration policy index 2020. Technical report, Barcelona Centre for International Affairs and Migration Policy Group, Barcelona and Brussels, 2020. URL https://www.mipex.eu.

[17] Brantly Callaway and Pedro H. C. Sant'Anna. Difference-in-differences with multiple time periods. *Journal of Econometrics*, 225(2):200–230, 2021. doi: 10.1016/j.jeconom.2020.12.001.

[18] Licheng Liu, Ye Wang, and Yiqing Xu. A practical guide to counterfactual estimators for causal inference with time-series cross-sectional data. *American Journal of Political Science*, 68(1):160–176, 2024. doi: 10.1111/ajps.12723.

[19] T. D. Stanley and Hristos Doucouliagos. Meta-regression approximations to reduce publication selection bias. *Research Synthesis Methods*, 5(1):60–78, 2014. doi: 10.1002/jrsm.1095.

[20] Abel Brodeur, Nikolai Cook, and Anthony Heyes. Methods matter: P-hacking and publication bias in causal analysis in economics. *American Economic Review*, 110(11):3634–3660, 2020. doi: 10.1257/aer.20190687.

[21] Sergiy Pivnenko and Don DeVoretz. The recent economic performance of Ukrainian immigrants in Canada and the US. IZA Discussion Paper 913, IZA, 2003.

[22] Don J. DeVoretz and Sergiy Pivnenko. The economic causes and consequences of Canadian citizenship. *Journal of International Migration and Integration*, 6(3-4):435–468, 2005. doi: 10.1007/s12134-005-1021-6.

[23] Barry R. Chiswick and Paul W. Miller. Language in the immigrant labor market. In Barry R. Chiswick, editor, *Immigration, Language and Ethnicity: Canada and the United States*, pages 229–296. American Enterprise Institute, Washington, DC, 1992.

[24] Ather H. Akbari. Immigrant naturalization and its impacts on immigrant labour market performance and treasury. In Pieter Bevelander and Don J. DeVoretz, editors, *The Economics of Citizenship*, pages 129–154. Malmö University (MIM), Malmö, 2008.

[25] John E. Hayfron. The economics of Norwegian citizenship. In Pieter Bevelander and Don J. DeVoretz, editors, *The Economics of Citizenship*, pages 91–104. Malmö University (MIM), Malmö, 2008.

[26] Martin Kahanec and Anzelika Zaiceva. Labor market outcomes of immigrants and non-citizens in the EU: An East-West comparison. *International Journal of Manpower*, 30(1-2):97–115, 2009. doi: 10.1108/01437720910948429.

[27] Francesca Mazzolari. Dual citizenship rights: Do they make more and richer citizens? *Demography*, 46(1):169–191, 2009. doi: 10.1353/dem.0.0038.

[28] Heidi Shierholz. The effects of citizenship on family income and poverty. Briefing Paper 256, Economic Policy Institute, 2010.

[29] Bernt Bratsberg and Oddbjørn Raaum. The labour market outcomes of naturalised citizens in Norway. In *Naturalisation: A Passport for the Better Integration of Immigrants?* OECD Publishing, Paris, 2011. doi: 10.1787/9789264099104-10-en.

[30] Mattias Engdahl. The impact of naturalisation on labour market outcomes in Sweden. In *Naturalisation: A Passport for the Better Integration of Immigrants?* OECD Publishing, Paris, 2011. doi: 10.1787/9789264099104-6-en.

[31] Max Friedrich Steinhardt and Jan Wedemeier. The labor market performance of naturalized immigrants in Switzerland—new findings from the Swiss labor force survey. *Journal of International Migration and Integration*, 13(2):223–242, 2012. doi: 10.1007/s12134-011-0213-5.

[32] Max Friedrich Steinhardt. Does citizenship matter? the economic impact of naturalizations in Germany. *Labour Economics*, 19(6):813–823, 2012. doi: 10.1016/j.labeco.2012.09.001.

[33] Huiquan Zhou and Sungkyu Lee. Effects of US citizenship on wages of Asian immigrant women. *International Journal of Social Welfare*, 22(4):420–430, 2013. doi: 10.1111/ijsw.12010.

[34] Mattias Engdahl. Naturalizations and the economic and social integration of immigrants. Working Paper 2014:11, Institute for Evaluation of Labour Market and Education Policy (IFAU), 2014.

[35] Ravi Pendakur and Pieter Bevelander. Citizenship, enclaves and earnings: comparing two cool countries. *Citizenship Studies*, 18(3-4):384–407, 2014. doi: 10.1080/13621025.2014.905279.

[36] Won Joong Kim, Yongseung Han, and Myeong Hwan Kim. Determinations of naturalization and its impact on earnings. *European Scientific Journal*, 11(7):209–228, 2015.

[37] Vahan Sargsyan. Treatment-related naturalization premiums in two European countries: Evaluation and comparison. Working Paper 585, CERGE-EI, 2017.

[38] Jan-Jan Soon. Flags and anthems: naturalisation effects on income and employment. *International Journal of Social Economics*, 44(4):491–504, 2017. doi: 10.1108/ijse-06-2015-0179.

[39] Christina Gathmann and Nicolas Keller. Access to citizenship and the economic assimilation of immigrants. *The Economic Journal*, 128(616):3141–3181, 2018. doi: 10.1111/ecoj.12546.

[40] Marco Pecoraro and Philippe Wanner. Do dual citizenship rights make more and healthier citizens? Evidence from a quasi-experiment. Annual Conference of the Italian Economic Association, Bologna, 2018.

[41] Daniel Crown and Alessandra Faggian. Naturalization and the productivity of foreign-born doctorates. *Journal of Geographical Systems*, 21(4):533–556, 2019. doi: 10.1007/s10109-019-00301-6.

[42] Jens Hainmueller, Dominik Hangartner, and Dalston Ward. The effect of citizenship on the long-term earnings of marginalized immigrants: Quasi-experimental evidence from Switzerland. *Science Advances*, 5(12):eaay1610, 2019. doi: 10.1126/sciadv.aay1610.

[43] Miao Chi and Michael Coon. Variations in naturalization premiums by country of origin. *Eastern Economic Journal*, 46(1): 102–125, 2020. doi: 10.1057/s41302-019-00149-0.

[44] Joachim Jarreau. Naturalization policy and the economic integration of immigrants: new evidence for France. Working paper, 2020.

[45] Floris Peters, Maarten Vink, and Hans Schmeets. Anticipating the citizenship premium: Before and after effects of immigrant naturalisation on employment. *Journal of Ethnic and Migration Studies*, 44(7):1051–1080, 2018. doi: 10.1080/1369183X.2017.1367650.

[46] Floris Peters, Hans Schmeets, and Maarten Vink. Naturalisation and immigrant earnings: Why and to whom citizenship matters. *European Journal of Population*, 36(3):511–545, 2020. doi: 10.1007/s10680-019-09540-1.

[47] Yajna Govind. Is naturalization a passport for better labor market integration? Evidence from a quasi-experimental setting. PSE Working Paper 2021-42, Paris School of Economics, 2021.

[48] Wladimir Zanoni and Ailin He. Citizenship and the economic assimilation of Canadian immigrants. IDB Working Paper Series IDB-WP-01127, Inter-American Development Bank, 2021.

[49] Davide Gritti, Filippo Gioachin, Anna Zamberlan, Eleonora Meli, Raffaele Grotti, Paolo Barbieri, and Stefani Scherer. Citizenship acquisition and labour market outcomes among immigrants in Italy: Evidence from linked survey-register data. *International Migration*, 63:e70069, 2025. doi: 10.1111/imig.70069.

[50] OECD. *Naturalisation: A Passport for the Better Integration of Immigrants?* OECD Publishing, Paris, 2011. doi: 10.1787/9789264099104-en.
